# Overexpression of *lipA* or *glpD_RuBisCO* in the *Synechocystis* sp. PCC 6803 Mutant Lacking the *Aas* Gene Enhances Free Fatty-Acid Secretion and Intracellular Lipid Accumulation

**DOI:** 10.3390/ijms222111468

**Published:** 2021-10-25

**Authors:** Kamonchanock Eungrasamee, Aran Incharoensakdi, Peter Lindblad, Saowarath Jantaro

**Affiliations:** 1 Laboratory of Cyanobacterial Biotechnology, Department of Biochemistry, Faculty of Science, Chulalongkorn University, Bangkok 10330, Thailand; kamonchanock.eungrasamee@gmail.com (K.E.); Aran.i@chula.ac.th (A.I.); 2 Microbial Chemistry, Department of Chemistry–Ångström, Uppsala University, Box 523, SE-75120 Uppsala, Sweden; Peter.Lindblad@kemi.uu.se

**Keywords:** FFA secretion, acyl-ACP synthetase, lipase A, *Synechocystis* sp. PCC 6803, membrane lipid degradation

## Abstract

Although engineered cyanobacteria for the production of lipids and fatty acids (FAs) are intelligently used as sustainable biofuel resources, intracellularly overproduced FAs disturb cellular homeostasis and eventually generate lethal toxicity. In order to improve their production by enhancing FFAs secretion into a medium, we constructed three engineered *Synechocystis* 6803 strains including KA (a mutant lacking the *aas* gene), KAOL (KA overexpressing *lipA*, encoding lipase A in membrane lipid hydrolysis), and KAOGR (KA overexpressing quadruple *glpD*/*rbcLXS*, related to the CBB cycle). Certain contents of intracellular lipids and secreted FFAs of all engineered strains were higher than those of the wild type. Remarkably, the KAOL strain attained the highest level of secreted FFAs by about 21.9%w/DCW at day 5 of normal BG_11_ cultivation, with a higher growth rate and shorter doubling time. TEM images provided crucial evidence on the morphological changes of the KAOL strain, which accumulated abundant droplets on regions of thylakoid membranes throughout the cell when compared with wild type. On the other hand, BG_11_-N condition significantly induced contents of both intracellular lipids and secreted FFAs of the KAOL strain up to 37.2 and 24.5%w/DCW, respectively, within 5 days. Then, for the first time, we shone a spotlight onto the overexpression of *lipA* in the *aas* mutant of *Synechocystis* as another potential strategy to achieve higher FFAs secretion with sustainable growth.

## 1. Introduction

Photosynthetic cyanobacteria and microalgae are classified as third-generation renewable energy resources of biofuels, such as biodiesel, bio-oil, and biohydrogen, due to their atmospheric CO_2_ fixation without food source competition [1,2,3]. In cyanobacterial biomass, lipids and fatty acid (FAs) biomolecules are mainly distributed as components of the membranes [3]. In order to balance lipid homeostasis in the cyanobacterium *Synechocystis* sp. PCC 6803, not only FA and membrane lipid syntheses but also FFAs recycling and FFAs secretion are directly involved [4]. Increasing lipid-producing microorganisms by genetic engineering has gained attraction in recent years [4,5,6], but with limitations on productivity, such as toxicity of overproduced FAs. Excessive levels of intracellular FFAs in the engineered FFA-producing *Synechococcus elongatus* strain (dAS1T) ultimately generated toxicity and resulted in cell death [7]. Recently, to address the FFAs toxicity, *Synechocystis* sp. PCC6803, *Synechococcus elongatus* PCC7942, and *Synechococcus* sp. PCC 7002 strains secreting FFAs out of cells and into the culture medium have been identified [4,8,9,10]. This FFA-secretion property of cyanobacteria is considered an important aspect of industrial productivity, such as cost reduction of production and downstream processing, including harvesting, drying, and cell breakage for FFA product recovery [11]. Strategies for improving intracellular lipid production and FFA excretion from cyanobacteria and algae have recently been focused on physiological conditions (such as nitrogen and phosphorous deprivations), environmental stress challenges, and genetically metabolic engineering approaches [12,13,14]. Known mechanisms using metabolic engineering approaches for increasing FFA secretion in cyanobacteria were previously reported, such as weakening of cell membrane layers by manipulating the surface layer (S-layer) protein encoded by the *sll1951* gene in *Synechocystis* 6803 [8]. This allowed FFA diffusion across the lipid layers with inactivation of acyl-ACP synthetase (*aas*) which prevented FFA recycling to acyl-ACP, and as a result, a secretion of produced FFAs into the culture medium [4,15,16,17]. Heterologous expression of the *tesA* gene encoding thioesterase cleaves the thioester bond of acyl-ACP to FFAs [8,18]. Moreover, integrated strategies were extensively implemented to attain higher FFAs secretion, such as a modified strain with both *aas* inactivation and *tesA* expression [7,9,19,20], double *aac*/*tesA* expression integrated with an inactivation of *fadD* gene encoding fatty acyl-CoA synthase in *Escherichia coli* [21,22], or deletions of genes involved in acyl-CoA synthesis (such as *Δfaa* genes) and beta-oxidation (such as *Δpox1*) in *Saccharomyces cerevisiae* [23,24].

**Figure 1:**
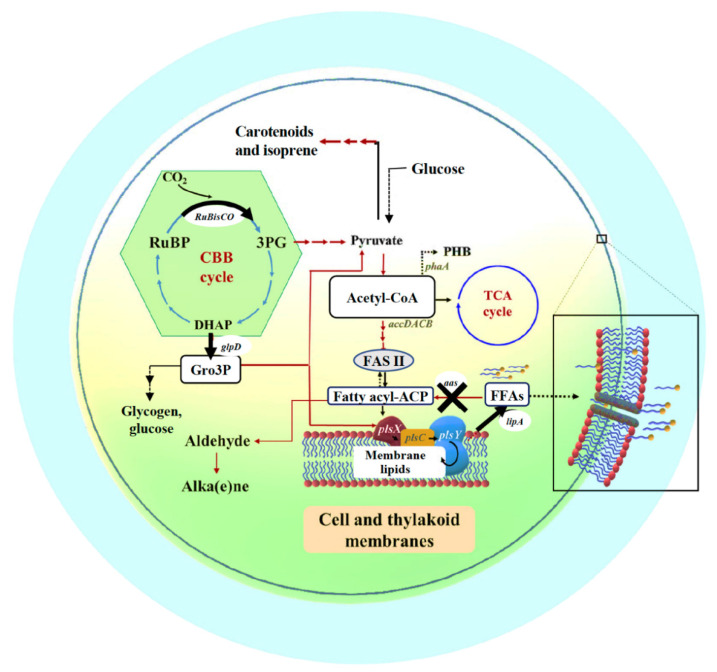
Outline of intracellular lipid production and FFA secretion into cultured medium system in cyanobacteria. Key enzyme-encoding genes (represented in italic letters) associated with lipid and fatty acid synthesis, the Calvin–Benson–Bassham (CBB) cycle, polyhydroxybutyrate (PHB) synthesis, and free fatty acid (FFA) recycling, including *accDACB*, a multi-subunit acetyl-CoA carboxylase gene; *lipA*, a lipolytic enzyme-encoding gene; *aas*, acyl-ACP synthetase gene; *plsX*, *plsY* and *plsC*, putative phosphate acyl-transferases genes; and *phaA*, acetyl-CoA acetyltransferase gene; the *RuBisCO* gene cluster, including *rbcLXS*, encoding RuBisCO in respective order of large, chaperone and small subunits; *glpD*, glycerol-3-phosphate dehydrogenase gene. The abbreviated intermediates and products are: DHAP, dihydroxyacetone phosphate; fatty acyl-ACP, fatty acyl–acyl carrier protein; FFAs, free fatty acids; Gro3P, glycerol-3-phosphate; 3PG, 3-phosphoglycerate; PHB, polyhydroxybutyrate; RuBP, ribulose-1,5-bisphosphate. The thick arrows represent the overexpression (O) of indicated genes whereas the cross symbol represents knockout (K) of indicated gene.

The metabolic networks of the Calvin–Bassham–Benson (CBB) cycle, fatty acid and membrane lipid biosyntheses and their linkage pathways including membrane hydrolysis and FFA recycling are depicted in Figure 1 (modified from [6]). In photosynthetic organisms, the CBB cycle, initially catalyzed by the key enzyme of RuBisCO, ribulose-1,5-bisphosphate carboxylase/oxygenase, provides many key intermediates, such as 3PG which is subsequently converted to pyruvate, and DHAP that flows through several reactions for RuBP production and partly to Gro3P via glpD (glycerol-3-phosphate dehydrogenase) catalysis [25,26]. This Gro3P, or glycerol-3-phosphate intermediate, is subsequently incorporated to many biochemical reactions, such as pyruvate production, gluconeogenesis, membrane lipid synthesis, and lycopene production [26,27]. For fatty acid and membrane lipid syntheses, acetyl-CoA is converted to malonyl-CoA, catalyzed by multi-subunit acetyl-CoA carboxylase, encoded by *accDACB*, and consecutively flows through fatty acid synthesis II (FASII) which may generate an intermediate fatty acyl–acyl carrier protein (fatty acyl-ACP), a substrate for membrane lipid synthesis via PlsX, PlsC and PlsY enzymes. The linkage between membrane lipids and the CBB cycle attains the glycerol backbone of lipid molecule from Gro3P, a product from glpD reaction. In terms of membrane lipid homeostasis, not only its synthesis but also degradation is substantially balanced. Final products, namely intracellular FFAs, from membrane lipid degradation or hydrolysis, are produced by lipase A catalysis encoded by *lipA* or *sll1969* in *Synechocystis* 6803 [16]. In addition, the intracellular FFA level is regulated to lower its toxicity to the cells by both the FFAs recycling reaction catalyzed by fatty acyl-ACP synthetase (encoded by *aas* or *slr1609* in *Synechocystis* 6803) for generating fatty acyl-ACP, and FFAs secretion [4,6]. On the other flow direction of acetyl-CoA (Figure 1), a synthesis of bioplastic polyhydroxybutyrate (or PHB) is inducible via acetyl-CoA acetyltransferase (*phaA*) catalysis and accumulated as neutral lipid granules in *Synechocystis*, in particular under nutrient deprived-conditions [28,29].

In this study, we performed an integrative design of three engineered strains of *Synechocystis* sp. PCC 6803. (1) KA strain, the disruption of the *aas* gene encoding acyl-ACP synthetase that blocks FFAs recycling, (2) KAOL strain, KA with the overexpression of *lipA* which increases the membrane lipid degradation, and (3) KAOGR strain, KA with the overexpression of the RuBisCO operon (*rbcL*, *rbcX* and *rbcS*) and *glpD* encoding glycerol-3-phosphate dehydrogenase, which enhance the carbon supply from the CBB cycle via Gro3P generation for fatty-acid and membrane-lipid productions (Figure 1). We reported that all single and integrated KA strains secreted a higher content of extracellular FFAs when compared to that by wild-type cells, in particular the KAOL strain has an increased production.

## 2. Results and Discussion

### 2.1. Engineered Constructs of Synechocystis sp. PCC 6803 Lacking aas (KA), and Its Hybrids with Single Overexpression of LipA (KAOL) and Quadruple Overexpression of glpD/rbcLXS (KAOGR)

Our results showed that the inactivation of acyl-ACP synthetase (*aas*), which prevented FFA recycling to fatty acyl-ACP, secreted more free fatty acids (FFAs) into the culture medium, in agreement with earlier reports in cyanobacteria [15,16,17]. Then, the *aas*-overexpression in *Synechocystis* sp. PCC 6803 (hereafter, *Synechocystis*) conversely resulted in a significant decrease of secreted FFAs content [6]. In the current study, we then aimed to improve the FFA secretion by creating *Synechocystis* cells lacking *aas* (or KA) as a base for two more constructs. We examined the impact of either directly elevated FFAs levels via the increased membrane lipid hydrolysis by single *lipA*-overexpression in the KA strain (or the KAOL strain), and the indirect elevated carbon-substrate supply via the CBB cycle for FFA synthesis by quadruple *GlpD*/*rbcLXS* overexpression in the KA strain (KAOGR) thereby increasing the FFA secretion into the surrounding medium (Figure 1).

**Figure 2:**
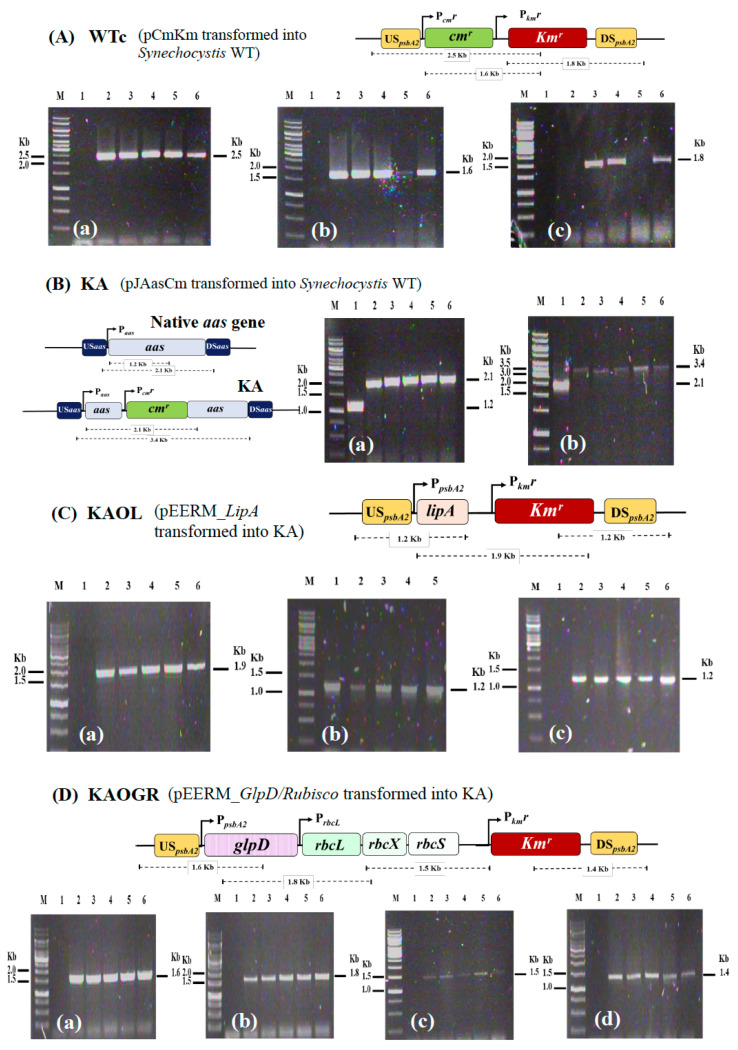
Genomic maps of engineered *Synechocystis* PCC 6803 strains. The four modified strains are WTc (**A**), KA (**B**), KAOL (**C**), and KAOGR (**D**). The specific primers (Supplementary Materials, Table S1) were used to confirm the integration of each gene into *Synechocystis* genome. WTc is a wild-type control made by transforming both antibiotic chloramphenicol and kanamycin resistance cassettes (*cm^r^* and *km^r^*, respectively) into WT strain. KA was constructed by inserting a *cm^r^* cassette to disrupt the *aas* gene. The integration of each strain was confirmed using PCR with genomic DNA from WT strain as the template. Lane M: GeneRuler DNA ladder (Fermentas). For (**A**) WTc strain; Lane 1: negative control using WTc as template (**a**–**c**), (**a**) Lanes 2–6: clone numbers 1 to 5 using UUSpsbA2 and Km_SR primers, (**b**) Lanes 2–6: clone numbers 1 to 5 using Cm_F and Km_SR primers, and (**c**) Lanes 2–6: clone numbers 1 to 5 using Km_FBamHI and DDSpsbA2 primers. For (**B**) KA strain; Lane 1: negative control using WTc as template (**a**,**b**), (**a**) Lanes 2–6: clone numbers 1 to 5 using Aas_F3 and aas_SR primers, and (**b**) Lanes 2–6: clone numbers 1 to 5 using USaas and DSaas primers. Only positive clones were selected for subsequent experiments; (**A**) WTc: clone numbers 2, 3, 5, (**B**) KA: clone numbers 1, 3, 4, 5, (**C**) KAOL: clone numbers 1–4, and (**D**) KAOGR: clone numbers 1–5.

We initially constructed a *Synechocystis* strain lacking *aas* or KA (Table 1). To gain reliable results concerning antibiotic *cm*^r^ and *km*^r^ effects, we constructed *Synechocystis* WT control (or WTc) by generating a pEERM_CmKm vector containing cassettes of *cm^r^* and *km^r^* which was consequently transformed into *Synechocystis* WT cells. Those cassettes of *cm^r^* and *km^r^* genes were integrated into *Synechocystis* WT genome at the native *psbA2* location via double homologous recombination (Figure 2A). The KA strain was constructed by inserting *cm^r^* cassette to disrupt the *aas* gene (Figure 2B). Moreover, the metabolic engineering approach was employed by integrating a recombinant plasmid containing *lipA* or *glpD*/*rbcLXS* genes to KA host strain, generating the KAOL and KAOGR strains, respectively. Those *lipA*- and *glpD*/*rbcLXS*-overexpressing strains were created by transforming pEERM_*LipA* and pEERM_*GlpD*/*rbcLXS* recombinants into KA host cells via double homologous recombination, and generated KA hybrid strains including the KAOL and KAOGR strains, respectively (Figure 2C,D). Then, its complete segregation was confirmed by PCR analysis using specific pairs of primers (Supplementary Materials, Table S1).

**Figure 3:**
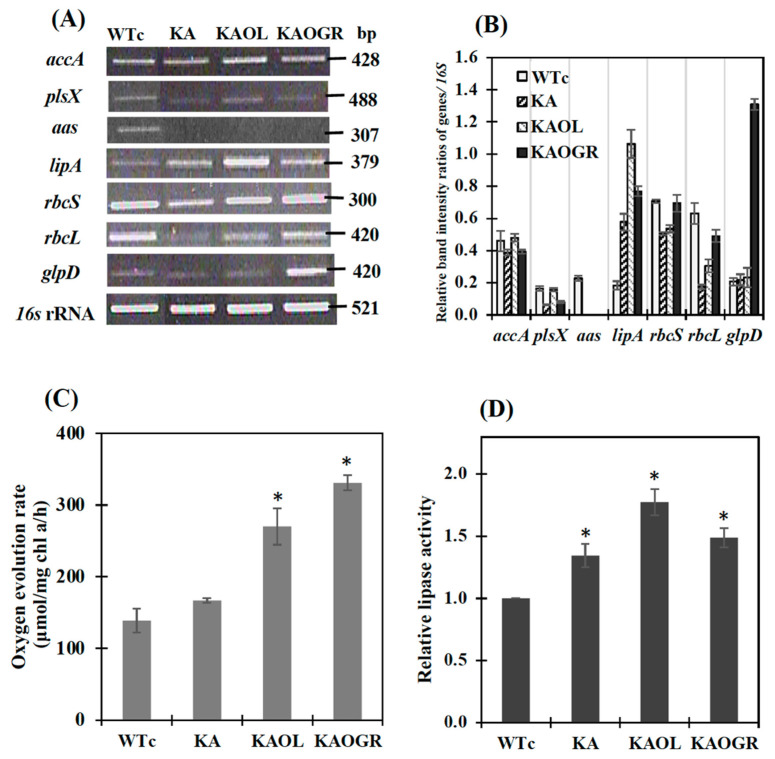
The transcript levels (**A**) and relative transcript intensity ratios (**B**) of the *accA*, *plsX*, *aas*, *lipA*, *rbcS*, *rbcL*, *glpD* and *16S* rRNA genes in *Synechocystis* PCC 6803 strains WTc, KA, KAOL and KAOGR. Oxygen evolution rate (**C**) and relative lipase activity (**D**) of all strains. Cells were grown in BG_11_ medium and analyzed at day 5 of cultivation. The error bars represent standard deviations of means (mean ± S.D., *n* = 3). For (**A**), agarose gel (1.2%) of PCR product stained by RedSafe^TM^ nucleic acid staining solution (Intron Biotechnology Inc., Korea). For (**D**), control data (WTc) at 1.0 is derived from the lipase activity of 0.22 U/mg protein. For (**C**) and (**D**), the statistical differences of the results between WTc and engineered strain are indicated by an asterisk *, *p* < 0.05.

We determined the relative transcript levels by using the RT-PCR method (Figure 3A,B). The noted transcript levels of *rbcS* and *rbcL* encoding RuBisCO in the CBB cycle were decreased in KA strain (Figure 3) when compared to those of WTc and of WT cells [4]. In the KAOL strain, the transcript level of *lipA* was clearly increased. Likewise, the apparent increases in *glpD*, *rbcS* and *rbcL* transcript levels were confirmed in the KAOGR strain. Moreover, a significant increase in the transcript levels of *accA*, *plsX*, *rbcS* and *rbcL* occurred in the KAOL strain when compared with those of KA host. In contrast, only a slight increase in *accA* and *plsX* transcript levels was observed in the KAOGR strain (Figure 3A). When compared with WTc, the noted decreases in *accA* and *plsX* transcript levels in KA and KAOGR suggested the reduced effect on fatty acid and phospholipid syntheses, while the increased effect on membrane hydrolysis occurred with higher *lipA* transcript levels (Figure 3B). As expected, a significant increase in the oxygen evolution rate, which represents photosynthetic efficiency, was observed in the KAOGR with overexpressing RuBisCO and KAOL strains compared to the WTc (Figure 3C). The highest level of lipase activity was found in the KAOL strain with *lipA* overexpression (Figure 3D), in agreement with its increased transcript level (Figure 3A,B).

**Figure 4:**
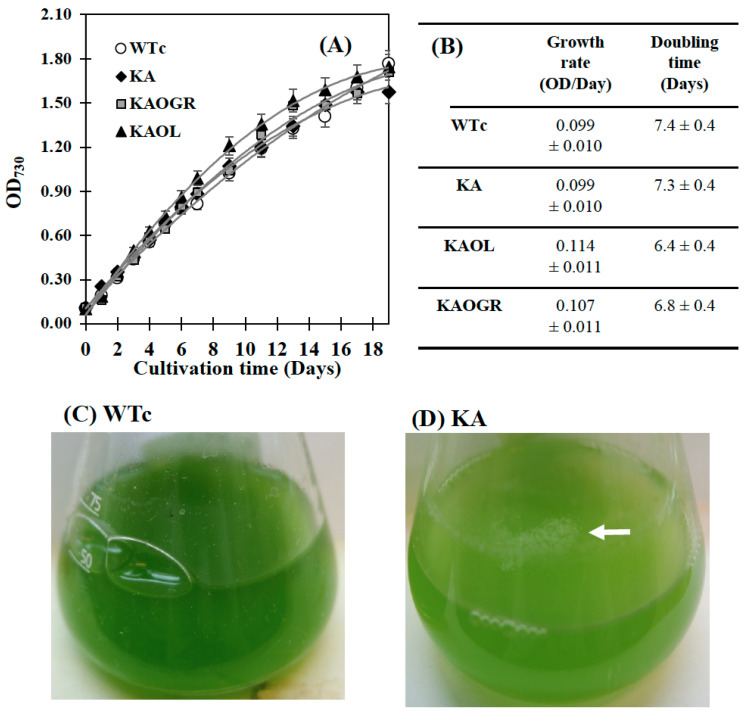

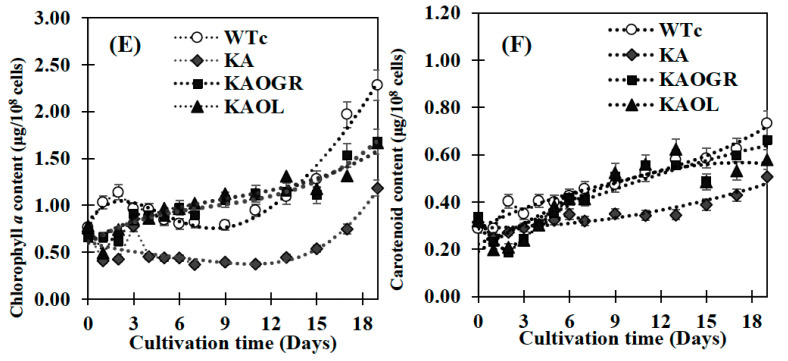
Growth curve (**A**), growth rate and doubling time (**B**), digital images of cell cultures (**C**,**D**), chlorophyll *a* content (**E**) and carotenoid content (**F**) of the *Synechocystis* PCC6803 wild-type control (WTc) and engineered KA strains. Cells were grown in BG_11_ medium for 19 days. Error bars represent standard deviations of means (mean ± S.D., *n* = 3). For (**C**,**D**), images of 10-day cell culture flasks of WTc and KA were depicted. Floating white droplets in KA-cultured flask represent free fatty acids (FFAs) secreted to the medium (white arrow).

The engineered WTc strain notably contained similar properties of growth and lipid production as WT cells [4]. Although the KA strain had a similar growth tendency to WTc, its intracellular pigment contents of chlorophyll *a* and carotenoids were markedly decreased when compared with WTc (Figure 4A–F). For qualitative results, digital images of the KA culture flask showed a lighter green color compared with the dark green of WTc cell cultures (Figure 4C,D). In addition, the white droplets floating on the surface of KA culture medium (Figure 4D), represented secreted FFAs, clearly visualized when compared with WTc without white droplets (Figure 4C). On the other hand, it was observed that the KA strain contained lower chlorophyll *a* and carotenoids accumulations than those of WTc cells (Figure 4E,F), in agreement with lowered transcript levels of *rbcS* and *rbcL* encoding RuBisCO enzyme in the CBB cycle (Figure 3A). We assume that RuBisCO substantially contributed to the accumulation patterns of chlorophyll *a* and carotenoids, in particular, carotenoids which relate to the pyruvate precursor partly converted from Gro3P ([27], and Figure 1). However, in respect to the lowered relative *rbcS* and r*bcL* transcript levels in the KA strain, although the oxygen evolution rate of the KA strain was similar to the WTc (Figure 3C), primary or secondary effects, such as RuBisCo activity or RuBP regeneration should be clarified for better understanding of its regulation. KAOGR, and specifically the KAOL strain, had slight increases in growth when compared with the WTc (Figure 4A), the latter clearly having a shorter doubling time (Figure 4B). However, the contents of chlorophyll *a* and carotenoids in the KAOL and KAOGR strains were slightly lower than in the WTc under normal conditions after 12 days of cultivation (Figure 4E,F).

### 2.2. Production of Intracellular Lipids qnd Extracellular FFAs in Synechocystis sp. PCC 6803 Wild-Type Control and Engineered Strains

**Figure 5:**
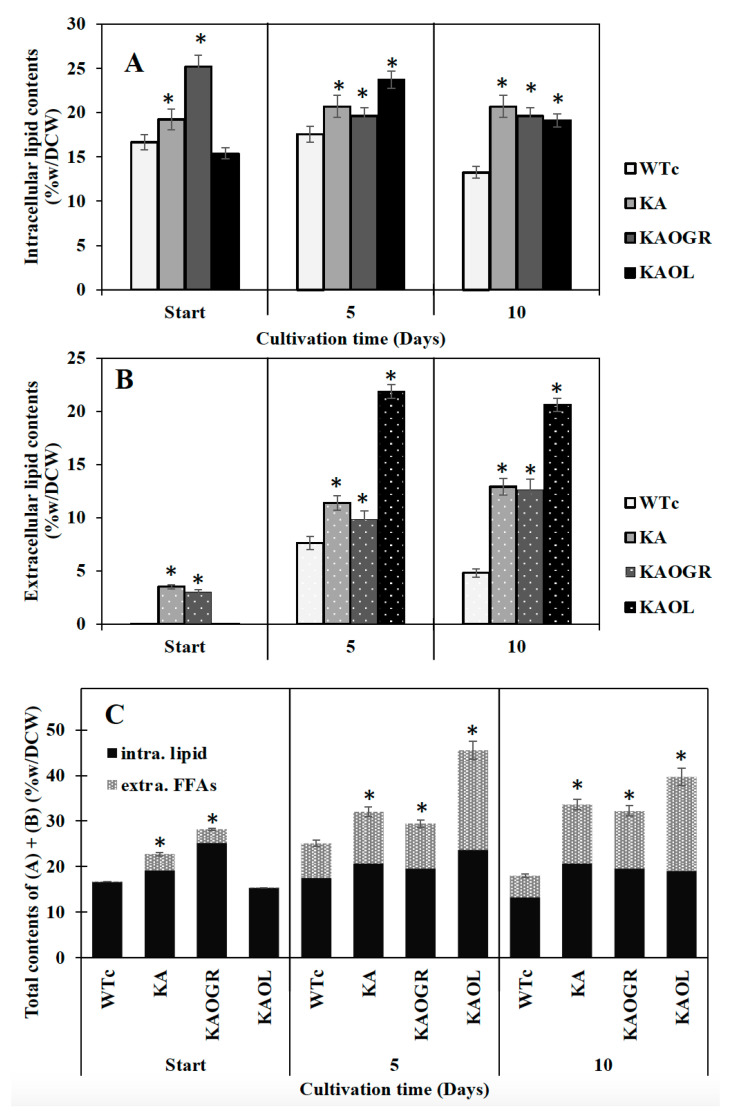
Contents (%w/DCW) of intracellular lipid (**A**) and extracellular FFAs (**B**) and total contents of intracellular lipid and extracellular FFAs (**C**) in *Synechocystis* PCC 6803 strains WTc, KA, KAOL and KAOGR. Cells were grown in BG_11_ medium at the start of the experiment (day 0) and analysed at days 5 and day 10 of cultivation. Error bars represent standard deviations of means (mean ± S.D., *n* = 3). The statistical differences of the results between the WTc and engineered strains are indicated by an asterisk *, *p* < 0.05.

Recently, radiolabeled FAs, thin layer chromatography (TLC) and gas chromatography (GC) have been employed to establish the intracellular and extracellular fractions of *Synechocystis* sp. PCC6803 containing FAs. A significant release of FFAs from membrane lipids to the surrounding culture medium was demonstrated [15]. Moreover, the white deposits or droplets of secreted FFAs in the culture medium of *Synechocystis* sp. PCC6803 were tangibly identified by GC/GC–MS [4,8,20]. In this study, although KAOGR showed the highest amount of intracellular lipid content at the start point, we experimentally ascertained that both intracellular lipid and extracellular FFAs contents of the KA strain were practically higher than those of the WTc by a constant lipid accumulation of about 20.7%w/DCW during 5–10 days of cultivation, whereas the extracellular FFA content reached up to 12.9%w/DCW at day 10 (Figure 5A,B). Consistent parameters of titer (mg/L) and production rate (mg/L/day) of KA strain were noted, corresponding to their respective contents (%w/DCW) of intracellular lipids and extracellular FFAs (Supplementary Materials, Table S2). Moreover, when compared among the three KA strains, the KAOL strain clearly contained the highest levels of both intracellular lipids, and showed the highest secreted FFAs at 5 days of cultivation, at 23.7 and 21.9%w/DCW, respectively (Figure 5A). This also holds for total contents of both intracellular lipids and extracellular FFAs (Figure 5C). Interestingly, the titer and production rates of the KAOL strain resulted in the highest levels of extracellular FFAs fractions at days 5 and 10 of cultivation (Supplementary Materials, Table S2).

Recent observations on increased lipid synthesis have shown that high lipid content occurred during the exponential growth phase and remained steady or decreased during the late-log and stationary phases [4,30]. The interplay of many biochemical pathways to maintain lipid homeostasis includes fatty acid and membrane lipid synthases, membrane lipid degradation, FFA recycling and FFA secretion. Most lipid molecules in cyanobacteria consist of four major types including monogalactosyl diacylglycerol (MGDG), galactosyl diacylglycerol (DGDG), sulfoquinovosyl diacylglycerol (SQDG) and phosphatidylglycerol (PG), located in both the cytoplasmic and thylakoid membranes [31]. Hence, membrane lipids could be hydrolyzed into free fatty acids (FFAs) by lipase A encoded by *sll1969* in *Synechocystis* PCC 6803 [16]. Those FFAs products are either partly secreted out of cells into the medium or are incorporated into membrane lipids’ biosynthesis via FFA recycling [32,33,34,35]. In the current study, we demonstrated that the *lipA* overexpression in the KA strain (KAOL) impacts the FFA secretion, with the highest level reaching 21.9%w/DCW at day 5 of cultivation (Figure 5), rather than that of KAOGR, a KA strain with overexpressing *glpD*/*rbcLXS* genes. Therefore, we reason that the regulation of FFA secretion may have a direct correlation with intracellular FFA yields, obtained either from the degradation of membrane lipids or the disturbance of FFAs recycling. This is in agreement with a previous strategy in which the prominent FFA secretion could be enhanced by weakening the cell-wall layers [8,36].

**Figure 6:**
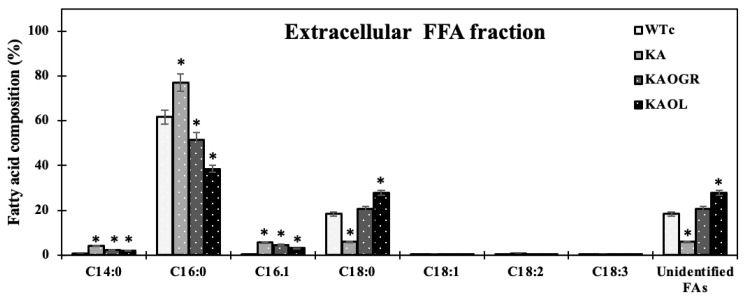
Fatty acid compositions (%) in extracellular fraction analysed by a GC instrument in *Synechocystis* PCC 6803 WTc and all engineered strains. Cells were grown in BG_11_ medium for 10 days before being analysed. Error bars represent standard deviations of means (mean ± S.D., *n* = 3). The statistical differences of the results between the WTc and engineered strains are indicated by an asterisk *, *p* < 0.05.

**Figure 7:**
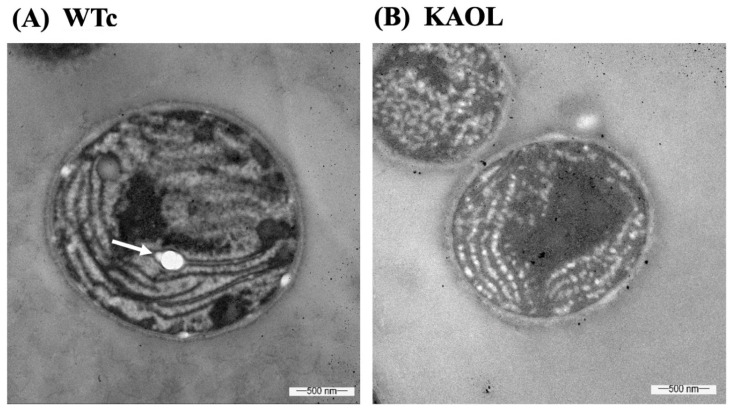
Transmission electron micrographs (TEMs) of *Synechocystis* WTc (**A**) and KAOL (**B**) strains under normal BG_11_ medium for 5 days of cultivation. In (**A**), WTc images are shown in 40,000× magnifications. In (**B**), KAOL images are shown in 50,000× magnifications. In (**A**), the white dots (white arrow) represent the examples of electron-transparent bodies. In (**B**), abundant droplet-like white dots were located on regions of thylakoid membranes inside KAOL cells.

Moreover, we also measured fatty acid (FA) compositions of extracellular FFA fraction by GC. For the KA strain, higher FA proportions were noted for myristic acid (C14:0), palmitic acid (C16:0), and palmitoleic acid (C16:1) (Figure 6). With the different FA compositions in the extracellular fraction of secreted FFAs, strains KAOL and KAOGR secreted higher proportions of stearic acid (C18:0), whereas a lower proportion of palmitic acid (C16:0) was noted when compared to both the KA and WTc strains. Interestingly, the relative contents of myristic acid (C14:0), palmitoleic acid (16:1), stearic acid (C18:0), and unidentified FAs, which may be longer than C18-Fas, were secreted into the medium in higher levels than those of intracellular FA pool. Certain structures of C16:0 and C18:0 molecules are more hydrophobic, which enabled them to more easily diffuse through cytoplasmic and thylakoid membranes [4]. The higher proportion of palmitoleic acid (16:1) in the extracellular FFA fraction was previously demonstrated for *Synechococcus elongatus* and *Synechocystis* sp. PCC 6803 [18]. For the qualitative images by TEM comparing the KAOL strain (Figure 7), the highest lipid producing strain, an abundant number of droplet-like bright dots located on regions of thylakoid membranes were visualized in the cells of the KAOL strain (Figure 7A,B). Interestingly, when we observed the cell membrane layers, the KAOL strain contained irregular and rough surfaces (Figure 7B) when compared with more regular and smooth cell surfaces of WTc cells (Figure 7A). We speculate that this rough surface of KAOL cells may support FFAs secretion.

**Figure 8:**
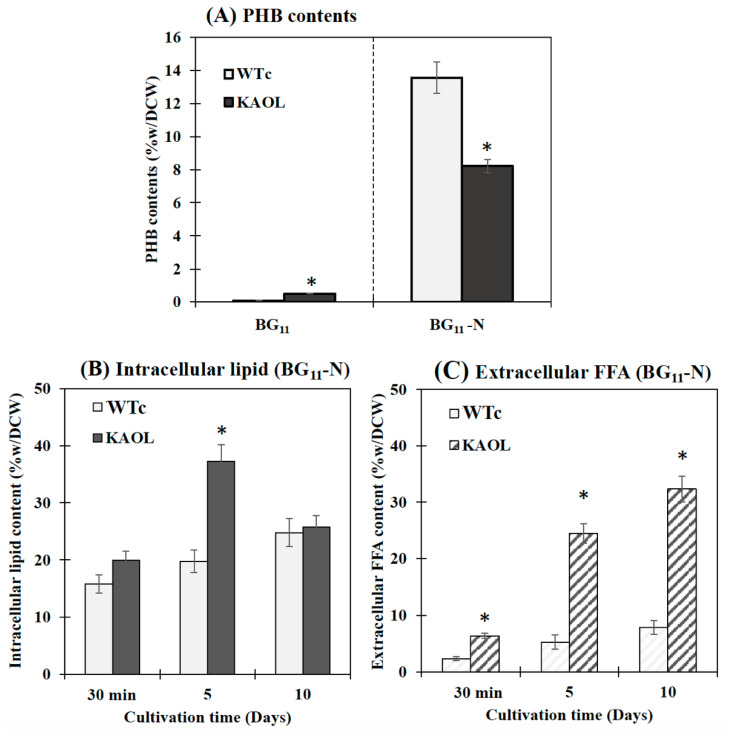
Contents (%w/DCW) of PHB (**A**), intracellular lipid (**B**) and extracellular FFAs (**C**) in *Synechocystis* PCC 6803 WTc and KAOL strains under BG_11_-N condition. For (**A**), cells were adapted for 5 days under BG_11_ and BG_11_-N conditions, respectively, before being analysed. For (**B**,**C**), cells were grown in BG_11_ medium lacking NaNO_3_ (BG_11_-N) at 30 min, day 5 and day 10 of cultivations. Error bars represent standard deviations of means (mean ± S.D., *n* = 3). The statistical difference of the results between the WTc and KAOL strains is indicated by an asterisk at * *p* < 0.05.

Excess of FFAs may cause toxicity and cell damage by intercalating their amphipathic structure into both cytoplasmic and thylakoid membranes [7,18]. As consequences of this, destroyed thylakoid pigments and destabilized membrane proteins detrimentally occur, as well as the disruption of the electron transport chain in photosynthesis of cyanobacteria [8,37,38]. Additionally, unsaturated fatty acids (UFAs), with double bonds, react with reactive oxygen species (ROS) and generate the toxic free-radical products which eventually damage the cell [39,40]. In this study, we observed that although the KA strain with FFA recycling blocked had similar growth and photosynthetic efficiency to the WTc, significant reductions in chlorophyll *a* and carotenoids occurred (Figure 4). These results may suggest that intracellular excess of FFAs which were not secreted out of cells can generate harmful effects on the intracellular pigments. However, *lipA*- and *glpD*/*rbcLXS*-overexpressions in KA strains, KAOL and KAOGR, may alleviate those negative consequences on the cell growth and retain regular pigment levels with higher photosynthetic efficiencies. Increased biomass yields of *Synechocystis* PCC 6803 were recently reported in the genetically modified strain, overexpressing either the *RuBisCO* operon [41], or *glpD_RuBisCO* [4] by generating increased carbon flow through the CBB cycle. On the other hand, it was surprising that the excess of FFAs in the KAOL strain had no negative effect on cell growth, oxygen evolution rate and intracellular pigment accumulations. Since there is no annotated fatty acid degradation pathway in *Synechocystis* [35], we propose that any excessive FFAs which are not secreted out of cells may be preferentially stored inside the cell by incorporation into higher intracellular lipids. Moreover, several droplet-like white granules can be identified inside the KAOL cells (Figure 7). Previously, an accumulation of lipid droplets, containing mainly saturated C16 and C18 fatty acids, was found in the cyanobacterium *Nostoc punctiforme*, induced when the cells consumed nitrate as their source of nitrogen during the log phase of cell growth [42]. We also demonstrated the polyhydroxybutyrate (PHB) contents and visualized Nile-red-stained PHB granules in both strains (Figure 8A and Supplementary Materials, Figure S1). It is worth noting that both WTc and KAOL cells contained less PHB granules under normal growth condition, visualized by fluorescence microscope (Supplementary Materials, Figure S1). This corresponds with a lower PHB content under normal BG_11_ conditions, detected by HPLC (Figure 8A). This obvious augmentation of droplet-like white spots then represents a crucial adaptation to significant membrane-lipids degradation, intracellularly generated by *lipA* overexpression.

### 2.3. NaNO_3_ Depletion Induces Higher Levels of Intracellular Lipids and Extracellular FFAs

It was noted that *Synechocystis* PCC 6803 WTc and KAOL cell cultures grown in BG_11_ lacking NaNO_3_ (or BG_11_-N) showed higher PHB production when compared to BG_11_ condition, demonstrated by an increased number of PHB granules (Supplementary Materials, Figure S1) and PHB content by about 13.6 and 8.2%w/DCW, respectively (Figure 8). This well-known strategy of nutrient deprivation was previously reported for PHB induction in *Synechocystis* sp. PCC 6803 [28,29]. However, it is also revealed that the KAOL strain showed lower PHB content than the WTc under BG_11_-N condition after 5 days (Figure 8A). We propose that this lower PHB content in the KAOL strain may partially inverse with a higher lipid content flow (see Figure 1). In short, KAOL cells showed higher contents of both intracellular lipids and extracellular FFAs under the BG_11_-N condition when compared to those observed in WTc cells (Figure 8B,C). The highest intracellular lipid level of the KAOL strain was 37.2%w/DCW at day 5, a 1.6-fold increase compared to that under BG_11_ condition (23.7%w/DCW). On the other hand, the secreted FFAs content of the KAOL strain significantly increased at days 5 and 10, reaching 24.5 and 32.3%w/DCW, respectively. This represents a 1.1- and 1.6-fold increase compared to those under BG_11_ condition (Figure 8C). Our findings support a conclusion that N-deprived conditions induce higher accumulations of intracellular lipids and secreted FFA in both *Synechocystis* wild-type and engineered strains, with overexpression of *lipA* in combination with a disruption of *aas*. Additionally, glycogen assimilation and degradation should be considered as competing for acetyl-CoA utilization, specifically in the KAOGR strain. An increased level of *glgX* encoding a glycogen debranching enzyme occurred in a high PHB-overproducing *Synechocystis* strain with overexpression of native *pha* gene [29].

## 3. Materials and Methods

### 3.1. Construction of Recombinant Plasmids

The recombinant plasmids, including pEERM_CmKm, pEERM_*GlpD*/*rbcLXS*, pJAasCm and pEERM_*lipA* (Table 1) were used to generate all strains including an *aas*-knock out *Synechocystis* (KA), *glpD*/*rbcLXS*-overexpressing KA (KAOGR) and *lipA*-overexpressing KA (KAOL). The recombinant pJAasCm vector was created by firstly ligating the *aas* gene fragment amplified by PCR with genomic DNA as a template, using aas_F3 and aas_R3 as primers (Supplementary Materials, Table S1) into the pJET1.2 blunt cloning vector. Secondly, the antibiotic *cm^r^* cassette fragment was amplified by cm_F and cm_R primers (Supplementary Materials, Table S1) using a pEERM plasmid [43] as the template. This *cm^r^* gene fragment was subsequently inserted onto the *Avr*II restriction site of the *aas* gene which earlier ligated into the pJET1.2 blunt cloning vector. On the other hand, the construction of the recombinant pEERM_*LipA* plasmid was performed by inserting the *lipA* gene fragment amplified by PCR using lipA_F and lipA_R as primers (Supplementary Materials, Table S1) inbetween the restriction sites of *Xba*I and *Spe*I in the pEERM_Km vector [4]. After that, the recombinant plasmids were confirmed by sequencing using specific primers as shown in Supplementary Materials, Table S1.

### 3.2. Natural Transformation of Recombinant Plasmids into Synechocystis Cells

Initially, the recombinant pEERM_CmKm and pJAasCm plasmids were separately transformed into the *Synechocystis* sp. PCC 6803 wild-type (WT) strain by natural transformation [6] in order to obtain the control WT (WTc) and an *aas*-knockout (KA) strains, respectively. Those WTc and KA strains were subsequently used as host strains. For pEERM_*glpD*/*rbcLXS* and pEERM_*lipA* recombinant plasmids, they were separately transformed into the KA host strain and finally the KAOGR and KAOL strains were obtained, respectively. The host cells were cultured in fresh BG_11_ medium and grown until the optical density at 730 nm (OD730) was about 0.3–0.5. Then, 50 mL of cell culture was harvested by centrifugation at 6000 rpm (4025 × *g*) for 10 min, and cell pellets were collected. To condense the cell suspension, fresh BG_11_ medium (0.5 mL) was subsequently added to suspended cell pellets. Next, 10 µg of recombinant plasmid was added into that condensed cell suspension and mixed gently by pipetting. The mixture was incubated at 28 °C for 6 h by inverting the mixture tube every 2 h under a continuous light intensity at 15 µE/m2s. After incubation, that mixture was smeared on a 0.45 µm sterile nitrocellulose membrane which was placed over a BG_11_ agar plate and incubated overnight, and then the membrane was transferred to be placed upon BG_11_ agar containing 35 µg/mL kanamycin, alone or combined with 35 µg/mL chloramphenicol. The surviving colonies were randomly picked from those plates after several weeks of incubating under normal growth conditions, and their gene locations and segregation were confirmed by PCR using specific pairs of primers, as shown in Supplementary Materials, Table S1.

### 3.3. Determinations of Cells Growth and Pigment Contents

*Synechocystis* WT and all engineered strains were initially cultivated on normal BG_11_ agar plate and selective BG_11_ agar plate containing antibiotic 35 µg/mL kanamycin and 35 µg/mL chloramphenicol, respectively. Normal growth condition was performed, at 28–32 °C under a continuous light illumination with an intensity of 40–50 µE/m^2^s. In order to prepare stock culture, colonies were transferred into a 250 mL-flask containing 100 mL of fresh liquid BG_11_ medium, and cultured until OD_730_ reaching 0.5–0.7 by shaking at 160 rpm on a rotary shaker. Cell culture was harvested by centrifugation at 6000 rpm (4025× *g*) for 10 min. Cell pellets were resuspended into fresh BG_11_ medium with initial OD_730_ at 0.1 and were subsequently cultured under normal growth condition for 16 days. One mL of cultured cells was collected to measure cell growth by spectrophotometrically measuring at OD730 [44]. For N-deprived conditions, cells at the mid-log phase of growth were harvested by centrifugation at 6000 rpm (4025× *g*) for 10 min and transferred into normal and modified media including normal BG_11_ medium (BG_11_ containing 17.6 mM NaNO_3_) and BG_11_ without NaNO_3_ (BG_11_-N). For pigment extraction, another 1 mL of cell culture was centrifuged at 6000 rpm (4025 g) for 10 min, and cell pellets were collected for pigment extraction using N,N-dimethylformamide (DMF). The DMF-extracted samples were subsequently measured for their absorbances at 461, 625 and 664 nm, respectively, by a spectrophotometer. The pigment contents were calculated and normalized by equations according to [45,46].

### 3.4. Lipid Extraction

During cultivation, 25 mL of cell culture of each strain was sampled and harvested by centrifugation at 6000 rpm (4025× *g*) for 10 min. The intracellular lipids and extracellular FFAs were separately obtained from cell pellets and supernatant fractions, respectively. The chloroform and methanol solvent extraction method was performed (modified from [47]). One mL of CHCL_3_: MeOH (ratio 2:1) solution was added into the cell pellet fraction whereas 5 mL of that solution was added into the supernatant fraction. The mixture was mixed by vortexing and incubated at 37 °C on a shaker for 2 h. Then, 0.5 mL of 0.88% *w*/*v* potassium chloride was added and the mixture was subsequently vortexed and left at room temperature for 5 min. The mixture was then centrifuged at 6000 rpm (4025× *g*) for 5 min, and the upper aqueous phase was removed, and the lower chloroform phase was collected and transferred to new glass tube. The lipid-containing chloroform phase was evaporated in fume hood at room temperature overnight to obtain total lipids.

### 3.5. Determinations of Intracellular Lipid and Extracellular FFA Contents

The extracted total lipids and secreted FFAs content were determined by the potassium dichromate oxidation reaction method [48]. Sequential additions of 0.18 M K_2_Cr_2_O_7_ (0.5 mL) and concentrated sulfuric acid (0.5 mL) were carried out and mixed with each sample by vortexing. The mixture was heated on a heat box at 100–105 °C for 30 min. After cooling down the reaction mixture to room temperature, distilled water (0.5 mL) was subsequently added. The absorbance of the reaction mixture was spectrophotometrically measured at 600 nm. Canola oil was used as a standard and prepared in the same way as the samples. The unit of lipid content was represented as % (w/DCW). Dried cell weight (DCW) was performed by drying the harvested cells in a 60–70 °C oven until obtaining a constant dry weight.

### 3.6. Determination of Fatty Acid (FA) Composition

Gas Chromatography (GC) was used for analyzing the fatty acid compositions from extracted lipids and FFAs in this study [4]. The fatty-acid methyl esters (FAMEs) were firstly prepared by adding 1 mL of 5%*v*/*v* hydrochloric acid in MeOH. The mixture reaction occurred by heating on a heat box at 85 °C for 2 h, and was then cooled down to room temperature. Distilled water (1 mL) was added and later vortexed for few seconds before adding 0.5 mL of hexane. In order to separate the reaction mixture, it was centrifuged at 6000 rpm (4025× *g*) for 5 min. The upper fraction of the hexane phase containing FAMEs was collected and transferred to GC vial tube. For analysis of fatty acid compositions, GC chromatograms were interpreted by comparing with standard equations. In this study, the commercial standard of fatty acid mixtures (F.A.M.E. mixed C8-C24 from SUPELCO©) was prepared in the same method as samples.

### 3.7. Total RNAs Extraction and Reverse Transcription-Polymerase Chain Reaction (RT-PCR)

Total RNAs were extracted from each strain using TRIzol^®^ Reagent (Invitrogen, Life Technologies Corporation, Carlsbad, CA, USA). The contaminated DNA was removed by treating with RNase-free DNaseI enzyme and DNaseI buffer before starting the reverse transcription step. The purified RNAs were calculated and further converted to cDNA using SuperScript™III First strand synthesis SuperMix Kit (Invitrogen, Carlsbad, CA, USA). After that, the cDNA of each stain was used as the template for PCR amplification of genes including *accA*, *plsX*, *aas*, *lipA*, *glpD*, *rbcS*, *rbcL* and *16S* rRNA (as a reference) by corresponding pairs of RT-PCR primers (Supplementary Materials, Table S1). The PCR conditions were sequentially performed by initial denaturing at 95 °C for 3 min, followed by suitable cycles of each gene (Supplementary Materials, Table S1) at 95 °C for 30 s, primer melting temperature (Tm, Supplementary Materials, Table S1) for 30 s and 72 °C for 30 s, and then final extension at 72 °C for 5 min. Then, the PCR products were checked by 1.2% agarose gel electrophoresis. Quantification of band intensity was detected by Syngene^®^ Gel Documentation (Syngene, Frederick, MD, USA).

### 3.8. Ultrastructure Visualization by Transmission Electron Microscopy (TEM)

The control WT (WTc) and engineered KAOL cells were visualized by transmission electron microscopy (TEM). Cells cultured in BG_11_ medium at day 5 of cultivation were harvested and sequentially fixed by adding 1 mL of the fixative solution containing 2.5% glutaraldehyde in 0.1 M phosphate buffer pH 7.0, and incubated at 4 °C for 2 h. After removing the fixative solution, 0.1 M phosphate buffer was added, and the reaction mixture was left at room temperature for 15–20 min. Next, the phosphate buffer was removed, and the cells were treated with 1% OsO_4_ in 0.1 M phosphate buffer for 1–2 h. The fixed pellets were washed by distilled water and subsequently coated with 2% agarose. After that, the coated cells were cut into blocks for dehydration and embedding. The embedded samples were again cut into sections with a thickness of about 80 nm by ultramicrotome and coated on the copper grid. These sections were stained with uranyl acetate and lead citrate solutions. The ultrastructure images were visualized by using transmission electron microscopy-JEM-1400Flash Electron Microscope operated at 80 kV.

### 3.9. Determination of PHB Contents by HPLC

Cell cultures (5–10 mL) of each condition were harvested by centrifugation at 6000 rpm (4025× *g*) for 10 min. To prepare the samples, 100 µL of 20 mg/mL of adipic acid (internal standard) and 800 µL of concentrated H_2_SO_4_ were added into cell pellets and further boiled at 100 °C for 1 h for digesting PHB to crotonic acid (modified from [29]). After that, 50 µL of the reaction mixture was diluted with 1.20 mL of ultrapure water. Then, 1 mL of solution was filtered through PP Syringe filter (0.45 µm, 13 mm), and collected in a glass vial for HPLC analysis (Shimadzu HPLC LGE System, Kyoto, Japan) using carbon-18 column with inert sustain of 3 µm (GL-Sciences, Tokyo, Japan). The running buffer was 30% (*v*/*v*) acetonitrile in 10 mM KH_2_PO_4_ at pH 2.3, with a flow rate of about 1.0 mL/min. The volume of crotonic acid was detected at 210 nm. Authentic commercial PHB (Sigma-Aldrich, Inc., St. Louis, MO, USA) as standard was prepared in the same way as the samples.

### 3.10. Fluorescence Microscopy of Nile-Red Stained Cells

Neutral lipids including PHB in *Synechocystis* cells were visualized by Nile-red staining dye (modified from [29]). Then, 100 µL of cell culture was harvested by centrifugation at 6000 rpm (4025× *g*) for 10 min. The cell pellets were washed by distilled water and stained by 30 µg/mL of Nile-red solution containing 0.9% (*w*/*v*) NaCl. The mixture was incubated in darkness overnight before observation under a fluorescent microscope (Olympus DP72, Tokyo, Japan), using a filter cube with 535 nm excitation wavelength.

### 3.11. Determination of Oxygen Evolution Rate

Five mL of cell culture was harvested by centrifugation at 6000 rpm (4025× *g*) for 10 min. The pellets were resuspended in 2 mL of fresh BG_11_ medium and incubated in darkness for 30 min. Thereafter, oxygen evolution of cells was measured under saturated light illumination by a Clark-type oxygen electrode (Hansatech instruments Ltd., King’s Lynn, UK) at 25 °C [6]. The oxygen evolution rate is presented as µmol/mg chlorophyll *a*/h.

### 3.12. Protein Extraction and Determination of Lipase Activity

To obtain crude protein, cell harvesting (20 mL culture) was carried out by centrifugation at 6000 rpm (4025× *g*), 4 °C for 10 min followed by cell extraction. Then, 200 µL of TB buffer (50 mM HEPES-NaOH (pH 7.0), 5 mM MgCl_2_, 25 mM CaCl_2_ and 10% (*v*/*v*) glycerol) was added to resuspend the cell pellets. Thereafter, glass beads were added into the cell suspension and the mixture was vigorously vortexed for 30 s and placed on ice for 15 s, repeated at least two times. After centrifugation at 6000 rpm (4025× *g*), at 4 °C for 5 min, the supernatant containing extracted proteins was transferred to a new tube. All supernatant fractions were pooled and subsequently centrifuged at 12,000 rpm (16,099× *g*), at 4 °C for 30 min to completely remove particles. The protein content was analyzed by the Bradford method [49] using commercial Sigma Aldrich^TM^ Bovine Serum Albumin, BSA as standard.

Lipase activity was determined by measuring the amount of *p*-nitrophenol (*p-*NP) released from *p-*NP ester substrate (modified from [50,51]). Crude extracted protein (20 μg) was added to 50 mM NaOAc (pH 8.0) containing 0.5 mM *p*-nitrophenylpalmitate (*p*-NPP), 10 mM isopropanol and 0.1% (*v*/*v*) Triton-X100. The reaction mixture was incubated at 37 °C for 60 min. The release of *p-*NP was measured spectrophotometrically at 405 nm. One unit of enzyme activity was defined as the amount of enzyme releasing 1 μmol of *p-*NP per minute.

## 4. Conclusions

Given the metabolic engineering approach that relies on FFA recycling, degradation of membrane lipids, and CBB cycle for improving FFA secretion of *Synechocystis* sp. PCC 6803, our findings from this study are expected to have broader implementations. We successfully constructed three engineered cyanobacterial strains including KA, KAOL and KAOGR strains, all with significantly increased levels of intracellular lipids and secreted FFAs, in particular the KAOL strain. The KAOL strain, a *lipA*-overexpressing KA, contained abundant white droplets and showed both distinct intracellular lipid production and FFA secretion, with a growth rate and an oxygen evolution rate higher than that of WT control cells. This novel, FFA-producing, cell factory continuously secretes fatty acids, and is a highly promising applied biotechnological finding, advancing the bioenergy research field.

## Figures and Tables

**Table 1:** Strains and plasmids used in this study.

Name	Relevant Genotype	Reference
**Cyanobacterial strains**		
*Synechocystis* sp. PCC 6803	Wild type	Pasteur Culture Collection
Control WT (WTc)	*cm^r^* and *km^r^* integrated at region of native *psbA2* gene in *Synechocystis* genome	This study
KA	*cm^r^* integrated at region of native *aas* gene in *Synechocystis* genome	This study
KAOL	*cm^r^* integrated at region of native *aas* gene in *Synechocystis* genome *lipA*, *km^r^* integrated at region of native *psbA2* gene in *Synechocystis* genome	This study
KAOGR	*cm^r^* integrated at region of native *aas* gene in *Synechocystis* genome *glpD*, *Rubisco*; *rbcL*, *rbcX*, *rbcS*, *km^r^* integrated at region of native *Rubisco* gene in *Synechocystis* genome	This study
**Plasmids**		
pEERM_CmKm	P_km_^r^–*km^r^*; integrated at BamHI sites of pEERM	This study
pEERM_Km	P_psbA2_–*km^r^*; plasmid containing flanking region of *psbA2* gene	[4]
pEERM_*GlpD*/*rbcLXS*	P_psbA2_–*glpD* and P*_rbcL_*-*rbcLXS*; integrated between *Xba*I*/Spe*I and *SpeI/PstI* sites of pEERM_Km, respectively	[4]
pEERM_*LipA*	P_psbA2_–*lipA*; integrated between *Xba*I and *Spe*I sites of pEERM_Km	This study
pJAasCm	P_T7_–*aas-cm^r^*; plasmid containing *cm^r^* between the flanking region of *aas* gene	This study

P_psbA2_, strong *psbA2* promoter; *cm^r^*, chloramphenicol resistance cassette; *km^r^*, kanamycin resistance cassette.

## Data Availability

Not applicable.

## Supplementary Materials

The following are available online at https://www.mdpi.com/article/10.3390/ijms222111468/s1.

## Abbreviations

AAS	acyl–acyl carrier protein synthetase
ACP	acyl carrier protein
Car	carotenoids
Chl *a*	chlorophyll *a*
CO_2_	carbon dioxide
DCW	dry cell weight
DMF	N,N-dimethylformamide
*E. coli*	Escherichia coli
FFA	free fatty acid
h	hour
lipA	lipase A
m	meter
μg	microgram
mL	milliliter
mg	milligram
min	minute
nm	nanometer
OD	optical density
PCR	polymerase chain reaction
plsX	putative acyltransferase
*p-*NP	*p-*nitrophenol
*p-*NPP	*p*-nitrophenylpalmitate
rbc	RuBisCO
rpm	revolutions per minute
s	second
TEM	transmission electron microscopy
WT	wild type
WTc	wild-type control
