# Supplementary material for: Overexpression of lipA or glpD_RuBisCO in the Synechocystis sp. PCC 6803 Mutant Lacking the Aas Gene Enhances Free Fatty-Acid Secretion and Intracellular Lipid Accumulation
Source: Int J Mol Sci. 2021 Oct 25;22(21):11468. doi: 10.3390/ijms222111468 (PMC8583886; doi:10.3390/ijms222111468)

Table S1 Primers used in this study

Name	Sequence (5' to 3')	Purpose of primer	PCR cycles /T_m	Expected size	Reference
Km_FBamHI	TAGAGAGGATCCACGGTTGATGAGAGCTTTGTTGTA	PCR for km^r		1122	This study
Km_FAclI	TAGAGAAACGTTACGGTTGATGAGAGCTTTGTTGTA	PCR for km^r			This study
Km_RBamHI	TAGAGAGGATCCTGTGTCTCAAAATCTCTGATGTTAC	PCR for km^r			This study
aas_F3	TAGAGATCTAGAGTGGACAGTGGCCATGGCGC	PCR for *aas*		2091	Eungrasamee et al. 2019
aas_R3	TACAGAACTAGTTTAAAACATTTCGTCAATTA	PCR for *aas*			Eungrasamee et al. 2019
lipA_F	TAGAGAACTAGTGTGGATTTTTTGACCAAGGG	PCR for *lipA*		615	This study
lipA_R	TAGAGACTGCAGTCAGTGGAGACAATAGTCGG	PCR for *lipA*			This study
cm_F	TAGAGACCTAGTCGAGTTGATCGGGCACGTAA	PCR for cm^r		905	This study
cm_R	TAGAGACCTAGTCAGCTCGAGGCTTGGATTCT	PCR for cm^r			This study
bb-cSR	GATATATCAACGGTGGTATATCCA	Sequencing			This study
RBC_SR0	AGTGACCTTTCATTTCGTTGGT	Sequencing			This study
RBC_SF1	ATGCGTTGGCGCGATCGTTTCC	Sequencing			This study
RBC_SR1	TTTGACAATTGCCAATGACTAA	Sequencing			This study
RBC_SF2	ACAGTTCTTCGGGGTTGAGTCT	Sequencing			This study
RBC_SR2	GGTTTGTGGACAATAAAGCTT	Sequencing			This study
GlpD_SR	GCGGTGGTCCGTGGTGCCAAT	Sequencing			This study
GlpD_SF	ATATGTGGAAGCGTTTGTGGATAAA	Sequencing			This study
Km_SF	CGTCATCAAAATCACTCGCA	Sequencing			This study
Km_SR	TGATGCATGGTTACTCACCACTGCG	Sequencing			This study
Km_R	TAGAGAGGATCCTGTGTCTCAAAATCTCTGATGTTAC	Colony PCR			Eungrasamee et al. 2020
UUSpsbA2	CACTCAGATAGGAGCCATCTTGC	Colony PCR			This study
USpsbA2	CTTTAGCGTTCCAGTGGATA	Colony PCR			This study
DSpsbA2	GCGATCGCCTTGGCAAAACAACTA	Colony PCR			This study
DDSpsbA2	CCCGTAGTTGTTCAATGATGATGAT	Colony PCR			This study
USrubisco	GCAACCCCTGATTAGCTTTGCG	Colony PCR			This study
DSrubisco	CCGGAATACTCCCCTGGGAA	Colony PCR			This study
USGlpD	CCTACTGCGGGAAGCCTACGCCG	Colony PCR			This study
DSGlpD	CTTAACCGTTGGCTATGGCGACAG	Colony PCR			This study
bb_f1	AGTTAGCCGTAGTTAGCCC	Colony PCR			This study
USaas (Aas_F3)	AGACAATCTAGAGTGGACAGTGGCCAT	Colony PCR			Eungrasamee et al. 2019
DSaas (Aas_R6)	ATAAACACTAGTTTAAAACATTTCGTC	Colony PCR			Eungrasamee et al. 2019
RTglpD_F420	GAATATGCGGAACGGTTAGAT	RT-PCR for *glpD*	26 cycles /53.5 °C	420	Eungrasamee et al. 2020
RTglpD_R420	GCGGTGGTCCGTGGTGCCAAT	RT-PCR for *glpD*			Eungrasamee et al. 2020
RTrbcL_F420	GGTTTTAAGGCTCTGCGGGC	RT-PCR for *rbcL*	23 cycles /58 °C	420	Eungrasamee et al. 2020
RTrbcL_R420	GATGATGGGGGTGCCAATTTCCT	RT-PCR for *rbcL*			Eungrasamee et al. 2020
RTrbcS_F300	CAAGCCGTTCTCAGGATCCAG	RT-PCR for *rbcS*	22 cycles /56 °C	300	Eungrasamee et al. 2020
RTrbcS_R300	AGGGAAATTATCGACTTCGGCTA	RT-PCR for *rbcS*			Eungrasamee et al. 2020
RTlipA_F379	TTGGCGGAGCAAGTGAAGCAAT	RT-PCR for *lipA*	25 cycles /55.1 °C	379	Eungrasamee et al. 2019
RTLipA_R379	CATGGACCAGCACAGGCAAAAT	RT-PCR for *lipA*			Eungrasamee et al. 2019
RTaccA_F428	ATGCACGGCGATCGAGGAGGT	RT-PCR for *accA*	27 cycles /58.2 °C	428	Eungrasamee et al. 2019
RTaccA_R428	TGGAGTAGCCACGGTGTACAC	RT-PCR for *accA*			Eungrasamee et al. 2019
RT16sRNA_F521	AGTTCTGACGGTACCTGATGA	RT-PCR for *16s*	13 cycles /56 °C	521	Eungrasamee et al. 2019
RT16sRNA_R521	GTCAAGCCTTGGTAAGGTTAT	RT-PCR for *16s*			Eungrasamee et al. 2019
RTaas_F307	GTGGTTTATCGCCGATCAAG	RT-PCR for *aas*	28 cycles /54.5 °C	307	Eungrasamee et al. 2019
RTaas_R307	TTCCTGGCGGGGAACGGGAG	RT-PCR for *aas*			Eungrasamee et al. 2019
RTPlsX_F	AAGGGGTGGTGGAAATGGAA	RT-PCR for *PlsX*	27 cycles /52.7 °C	488	Towijit et al. 2018
RTPlsX_R	AAGTAGGTCCCTTCCTTCGG	RT-PCR for *PlsX*			Towijit et al. 2018

Table S2 Titers and production rates of intracellular lipids and extracellular FFAs in all engineered strains of *Synechocystis* PCC 6803 compared with WTc (means ±S.D., n = 3). Cell pellet and supernatant fractions were harvested and separated by centrifugation at 6,000 rpm (4,025g) for 10 min. Lipid level of both pellet and supernatant fractions were extracted using dichromate oxidation method, and represented as intracellular lipids and extracellular FFAs, respectively. The statistical difference of the results between WTc and engineered strain is indicated by an asterisk at $*P < 0.05$.

**Strains**	**Intracellular Lipid titers (mg/L)**			**Production rates (mg/L/day)**	
	**Start**	**day 5**	**day 10**	**day 5**	**day 10**
**WTc**	8.9 ± 0.3	138.1 ± 10.1	148.4 ± 38.3	27.6 ± 5.2	14.8 ± 3.8
**KA**	11.4 ± 0.7*	146.3 ± 12.9	146.3 ± 12.7	29.3 ± 2.6	14.6 ± 1.3
**KAOL**	9.1 ± 0.3	148.4 ± 18.9	135.8 ± 1.8*	29.7 ± 3.8	13.6 ± 0.2*
**KAOGR**	14.9 ± 1.1*	145.9 ± 7.1*	145.9 ± 7.0*	29.2 ± 1.4	14.6 ± 0.7
**Strains**	**Extracellular FFA titers (mg/L)**			**Production rates (mg/L/day)**	
	**Start**	**day 5**	**day 10**	**day 5**	**day 10**
**WTc**	0.05 ± 0.02	9.0 ± 0.7	7.70 ± 1.6	1.80 ± 0.15	0.77 ± 0.16
**KA**	0.83 ± 0.03*	12.0 ± 0.5*	13.7 ± 2.8*	2.41± 0.11*	1.37 ± 0.28*
**KAOL**	0.05 ± 0.03	20.4 ± 1.1*	22.0 ± 0.8*	4.09 ± 0.23*	2.20 ± 0.08*
**KAOGR**	0.71 ± 0.04*	11.0 ± 0.2*	14.1 ± 1.0*	2.19 ± 0.05*	1.41 ± 0.10*

Figure S1 Images of *Synechocystis* 6803 wild type (A and C) and KAOL (B and D) adapted for 5 days under normal BG11 and BG11-N conditions, respectively. Images are from fluorescence microscopy showing PHB granules as bright gold particles with 100x magnification.

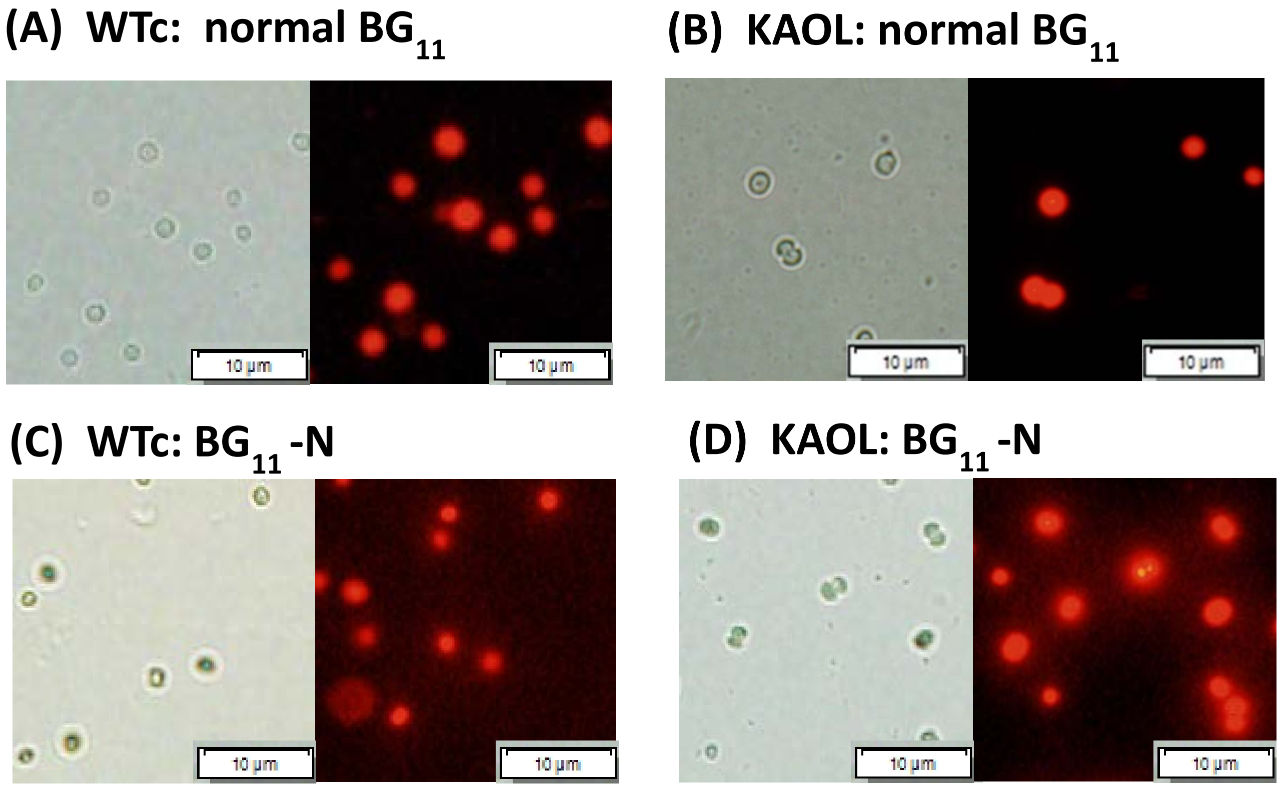

Supplement: Supplementary file 1 [file ijms-22-11468-s001.zip › ijms-1414368-supplementary.pdf]
